# Phylogenetic Lineages of *Francisella tularensis* in Animals

**DOI:** 10.3389/fcimb.2018.00258

**Published:** 2018-07-31

**Authors:** Paola Pilo

**Affiliations:** Vetsuisse Faculty, Institute of Veterinary Bacteriology, Department of Infectious Diseases and Pathobiology, University of Bern, Bern, Switzerland

**Keywords:** tularemia, animals, phylogenetic lineages, host specificity, ecology

## Abstract

Tularemia is a zoonotic disease caused by the facultative intracellular bacterium *Francisella tularensis*. This microorganism can infect a plethora of animal species and its ecology is particularly complex. Much research was performed to understand its biology but many questions are still open, especially concerning the life cycle of this bacterium in the environment related to physical and biological parameters. Numerous animals are major hosts of *F. tularensis* but precise reservoir species are not yet well defined. Moreover, the exact range of species susceptible to tularemia is not clear and is complicated by the differences in virulence and ecology observed among the subspecies of *F. tularensis*. Indeed, different life cycles in nature, including the animal species concerned, were previously described for *F. tularensis* subsp. *tularensis* and *F. tularensis* subsp. *holarctica*. Recently, molecular techniques showing adequate discrimination between strains were developed, leading to the possibility to investigate links between phylogenetic lineages and infection in animals. New perspectives in research are now possible thanks to the information available and the simplicity of the molecular procedures. Current studies are unfolding the evolution of *F. tularensis* and these developments will lead to the elucidation of geographical and ecological differences observed by veterinarians, microbiologists and conservation biologists. However, systematic, coordinated collection of data and extensive sampling are important to efficiently assemble the findings of future research.

## Introduction

Tularemia is a zoonotic disease known since the beginning of the last century. The disease was first described by McCoy in rodents in 1911 (McCoy, 1911) and the causative microorganism, *Francisella tularensis*, was later isolated from squirrels (McCoy and Chapin, 1912). The first bacteriologically confirmed human case description followed in 1914 (Wherry and Lamb, 1914). *F. tularensis* was subsequently isolated from hundreds of animal species (Mörner, 1992; WHO., 2007) and several arthropod vectors were identified (reviewed in Petersen et al., 2009). Because of the broad spectrum of potential vectors and hosts and the complex biology of the causative microorganism, the detailed understanding of the ecology of this bacterium is still unclear and many questions are still open regarding tularemia in animals, including which ones are incidental or reservoir species.

Tularemia occurs in parts of North America and Eurasia and seems to be restricted to the Northern Hemisphere. However, human and animal cases were recently described in Australia (Jackson et al., 2012; Eden et al., 2017). Over time, the geographic distribution and phenotypic characteristics including the virulence of *F. tularensis* strains appeared to be linked to the taxonomy of this bacterium and the species was divided into three clinically relevant subspecies: *tularensis* (type A), *holarctica* (type B), and *mediasiatica* (Olsufjev and Meshcheryakova, 1982; Eliasson et al., 2006). Briefly, the subspecies *tularensis* seemed to be confined to North America, showing high virulence to humans and animals. It is phenotypically characterized by its capacity to ferment glycerol and citrulline. Strains belonging to the subspecies *holarctica* were isolated from North America and Eurasia, displayed moderate virulence to humans and animals and were unable to ferment glycerol and citrulline. Interestingly, phenotypic variability was observed among these strains supporting a supplementary subdivision (Olsufjev and Meshcheryakova, 1982). The biotype “EryR” is erythromycin resistant, the biotype “EryS” is erythromycin sensitive and the biotype “japonica” is able to ferment glycerol. Subsequently, the subspecies *mediasiatica* was isolated from Central Asia and the strains exhibited moderate virulence but fermented glycerol and citrulline (Sjöstedt, 2015).

One of the first aspects considered by scientists to investigate the biology of *F. tularensis* was the variability in the degree of virulence of strains in experimental animal infections. Researchers rapidly described a severe and a mild form of tularemia among animals (Davis et al., 1934; Bell et al., 1955; Olsufjev and Meshcheryakova, 1982). Indeed, laboratory animals were used to isolate strains and to assess their degree of virulence (Davis et al., 1934; Philip and Davis, 1935). The quantitative measurement of virulence was mainly based on the amount of bacteria needed to kill the host and the number of days of survival after subcutaneous infection with a small number of bacteria. Later, Bell et al. (1955) standardized the protocols in mice, guinea pigs and rabbits. Furthermore, they confirmed a decreased sensitivity of rabbits compared to mice and guinea pigs to the strain 425F4G (*F. tularensis* subsp. *holarctica*). This observation and the phenotypic differences described above led to the confirmation of the taxonomic differentiation between *F. tularensis* subsp. *tularensis* and *F. tularensis* subsp. *holarctica* (Olsufjev and Meshcheryakova, 1982).

Although tularemia is described as potentially affecting hundreds of vertebrate species in natural settings, infections in lagomorphs and rodents are principally reported (Mörner, 1992). Moreover, isolated cases or outbreaks in captive primates, domestic cats and sheep are also documented. In animals, the clinical course of the disease appears to be dependent upon the susceptibility and sensitivity of the species (see section Tularemia in Animals of this review) (WHO., 2007). However, little information about clinical manifestations in naturally infected animals or the complete range of species affected is available.

The recent rapid development of sequencing methods allowed progress in the subdivision and typing of this microorganism. This is of major importance in the goal to dissect differences observed in ecology and epidemiology of *F. tularensis*. For these reasons, this review focuses principally on recent literature available on animal species naturally infected with this bacterium, excluding invertebrates, and highlights the data on genetic lineages associated with animal species.

## Genetic typing of strains of *F. tularensis*

**Figure 1:**
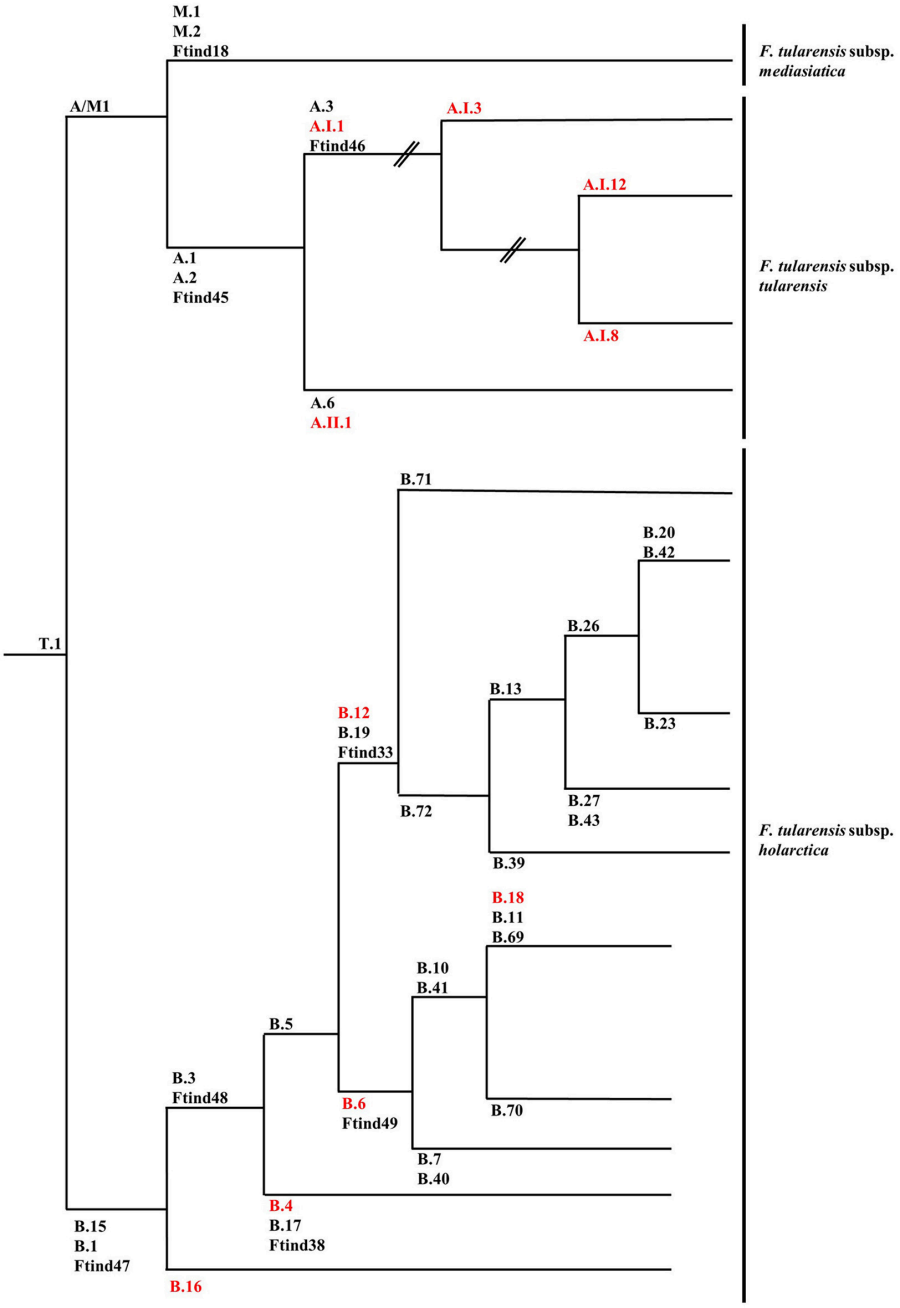
Schematic phylogenetic tree based on canSNP and INDELs of *Francisella tularensis* subsp. *tularensis, Francisella tularensis* subsp. *Holarctica*, and *Francisella tularensis* subsp. *mediasiatica*. When available, alternative marker for lineages are given and lineages discussed in the review are in red. Branch length is not representative of evolutionary distance and is not scaled. Data presented in this figure was collected and assembled from the following publications: (Svensson et al., 2009a,b; Vogler et al., 2009; Gyuranecz et al., 2012; Birdsell et al., 2014a,b; Sissonen et al., 2015).

**Figure 2:**
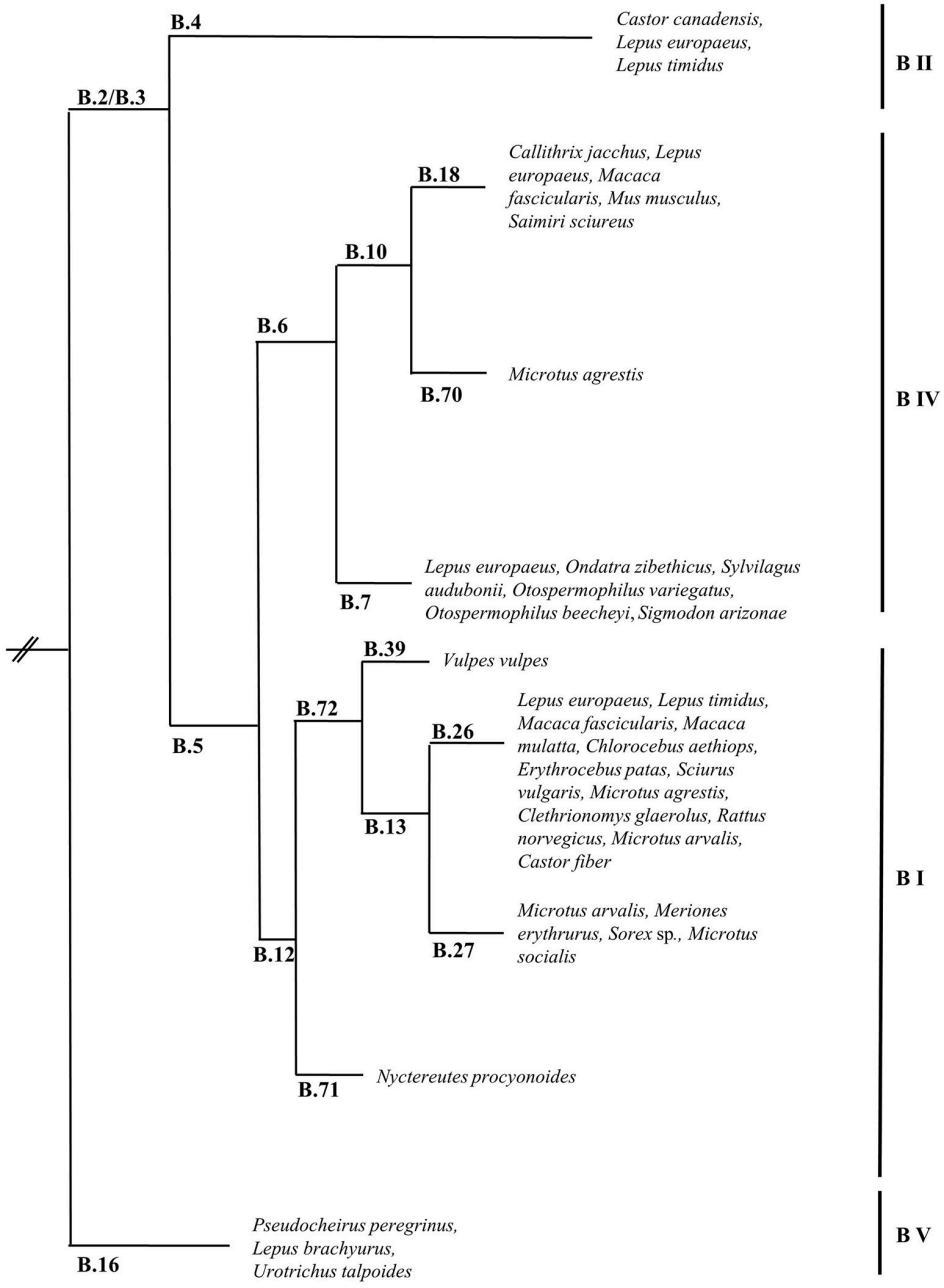
Schematic phylogenetic tree based on canSNP and INDELs of *Francisella tularensis* subsp. *holarctica* showing the major lineages and confirmed lineages of strains isolated from animal species (only of species specifically named in the literature). Branch length is not representative of evolutionary distance and is not scaled. Data presented in this figure was collected and assembled from the following publications: (Farlow et al., 2001; Abril et al., 2007; Fujita et al., 2008; Svensson et al., 2009b; Vogler et al., 2009; Chanturia et al., 2011; Gyuranecz et al., 2012; Müller et al., 2013; Origgi et al., 2014; Elashvili et al., 2015; Sissonen et al., 2015; Dwibedi et al., 2016; Myrtennas et al., 2016; Schulze et al., 2016; Eden et al., 2017).

The first attempts to type strains of *F. tularensis* using molecular approaches were hampered by the genetic homogeneity of the *F. tularensis* genome. Repetitive extragenic palindromic element PCR (REP-PCR), enterobacterial repetitive intergenic consensus sequence PCR (ERIC-PCR), random amplified polymorphic DNA (RAPD), pulsed-field gel electrophoresis (PFGE), and restriction fragment length polymorphism (RFLP) assays were assessed but gave little discrimination among strains or were troubled by reproducibility issues between laboratories (de la Puente-Redondo et al., 2000; Johansson et al., 2000; García Del Blanco et al., 2002; Thomas et al., 2003). The more recent development of a method based on the amplification of variable-number tandem repeats (VNTRs), which are fast evolving markers, allowed an adequate differentiation of strains and simplified comparisons between laboratories (Farlow et al., 2001; Johansson et al., 2001, 2004). The analysis of comprehensive collections of strains by multiple-loci VNTR analysis (MLVA) enabled the confirmation of the subdivision of *F. tularensis* subsp. *tularensis* in two major clades (A.I and A.II), while *F. tularensis* subsp. *holarctica* was separated into five clades (B.I, B.II, B.III, B.IV, and B.V) (Johansson et al., 2004). *F. tularensis* subsp. *mediasiatica* was recently divided into three clades: M.I, M.II, and M.III (Timofeev et al., 2017). However, bias inherent to the evolution of VNTRs does not permit solid phylogenetic studies since these markers are prone to homoplasy resulting from convergent evolution. To circumvent this drawback, assays based on canonical single nucleotide polymorphism (canSNP) and on canonical insertions/deletions (INDELs) were established (Figure 1) (Larsson et al., 2007; Vogler et al., 2009). As an example, the five clades initially described for *F. tularensis* subsp. *holarctica* were reduced to four clades (B.12 [B.I], B.4 [B.II], B.6 [B.IV] and B.16 [B.V]) because of inconsistent classification using MLVA (Figures 1, 2) (Svensson et al., 2009b; Karlsson et al., 2013). The development of these molecular tools for the typing of *F. tularensis* allowed not only better discrimination of strains and the possibility to perform population genetics and epidemiological studies but also to design rational panels of PCRs based on hierarchical schemes (Svensson et al., 2009b). These canSNP and INDEL systems were further applied to study worldwide collections of strains and new markers specific to distinct branches of the phylogenetic tree were identified. These improvements performed in the typing techniques of *F. tularensis* led to identify precise links between lineages and macrogeographical origin of strains, and to confirm the geographical overlapping of distinct subspecies and lineages. A large number of research groups investigated the distribution of the genetic diversity of strains in their respective countries. These studies led to the discovery of a very precise and vast amount of sublineages derived from basal lineages. As a consequence, the cladistic nomenclature was particularly detailed in the recent years (Chanturia et al., 2011; Hansen et al., 2011; Vogler et al., 2011; Gyuranecz et al., 2012; Karlsson et al., 2013; Müller et al., 2013; Origgi et al., 2014; Wang et al., 2014; Karadenizli et al., 2015; Kilic et al., 2015; Sissonen et al., 2015; Dwibedi et al., 2016; Myrtennas et al., 2016; Schulze et al., 2016).

The early population studies carried out using PFGE, MLVA, and canSNPs in North America uncovered novel information and new hypotheses concerning the biology and potential life cycle of *F. tularensis*. Firstly, strains of *F. tularensis* subsp. *tularensis* presented a higher genetic diversity than strains of *F. tularensis* subsp. *holarctica* suggesting a recent emergence of the subspecies *holarctica* (Johansson et al., 2004). Furthermore, particular lineages seemed to be associated with variation in virulence and potential animal host and vector specificity (Farlow et al., 2005; Staples et al., 2006; Kugeler et al., 2009). Molecular epidemiological investigations, using *Pme*I PFGE, of strains isolated in the USA showed differences in case fatality rates of tularemia in humans depending on lineages of *F. tularensis* subsp. *tularensis*; with A.Ia: 4%; A.Ib: 24%; AII: 0% and *F. tularensis* subsp. *holarctica* lineage B: 7% (Staples et al., 2006; Kugeler et al., 2009). This finding was later tested and experimentally confirmed in C57BL/6 mice (Molins et al., 2010). However, the clustering observed by PFGE typing is not completely compatible with the canSNP typing. Indeed, all A.Ia strains belong to the sublineage A.I.12 (successively tested by the canSNP method) but strains previously assigned to cluster A.Ib are disseminated in all the new identified sublineages: A.I.3, A.I.8, and A.I.12 (Figure 1). Concerning the potential virulence and ecological niches inhabited by the lineages of *F. tularensis* subsp. *holarctica*, less information is available. Specificities might be subtler for this subspecies than for *F. tularensis* subsp. *tularensis* due to the relative recent emergence of this subspecies resulting in low genetic heterogeneity. For *F. tularensis* subsp. *mediasiatica* there is very little data available on potential ecological niche differences among genetic lineages.

Links between *F. tularensis* lineages, virulence and niches are particularly interesting as several distinct life cycles related to ecological parameters have been described but the molecular mechanisms underlying these differences are still unknown. A well-defined differentiation and characterization of the lineages is central in order to expand knowledge in this field.

## Ecology of *F*. *tularensis*

Since the first description of tularemia and ensuing isolation of *F. tularensis*, several researchers investigated the host range, vectors and spread of this microorganism. Early on, it became clear that the epidemiology of tularemia is strictly connected to biological and physical features and an incredible diversity of ecological cycles of *F. tularensis* were designated. However, despite the large amount of research published on the ecology of *F. tularensis*, little is known about its life cycle in nature and the specific role of biological and physical parameters.

In North America, two major cycles were generally characterized: a terrestrial or sylvatic cycle and an aquatic cycle (Mörner, 1992). The terrestrial cycle mainly involves lagomorphs and more specifically *Sylvilagus* sp. and ticks, while the aquatic cycle comprises semi-aquatic rodents like the American beaver *Castor canadensis* and the muskrat *Ondatra zibethicus* (Mörner, 1992). It was later discovered that *F. tularensis* subsp. *tularensis* was involved in the sylvatic cycle and *F. tularensis* subsp. *holarctica* was isolated from animal species of the aquatic cycle.

In Eurasia, most of the studies were performed in former USSR and Scandinavia. In former USSR, ticks, rodent species like the water vole *Arvicola terrestris*, the common vole *Microtus arvalis*, the hamster *Cricetus* sp., the house mouse *Mus musculus* and lagomorphs, *Lepus* sp., are mainly affected by *F. tularensis* subsp. *holarctica* (Hopla, 1974). In Scandinavia, mosquitos, the mountain hares *Lepus timidus*, the European brown hare *L. europaeus*, the lemming *Lemmus lemmus* and the field vole *M. agrestis* are most frequently described as possibly contributing to the biological cycle of this bacterium (Hopla, 1974; Morner et al., 1988b; Rossow et al., 2014; Hestvik et al., 2017). In Eastern and Western Europe, *F. tularensis* subsp. *holarctica* is more often isolated or identified from ticks, the European brown hare *L. europaeus* and the common vole *M. arvalis* (de la Puente-Redondo et al., 2000; Kaysser et al., 2008; Gyuranecz et al., 2010; Decors et al., 2011; Origgi et al., 2014; Rodriguez-Pastor et al., 2017). In Japan, ticks and the Japanese hare *L. brachyurus* are confirmed hosts of *F. tularensis* subsp. *holarctica* (Fujita et al., 2008; Park et al., 2009). The host range of *F. tularensis* subsp. *mediasiatica*, isolated in Central Asia, is virtually unknown because of the very limited number of strains that have been isolated so far.

Beyond the animal species regularly identified as infected with *F. tularensis*, the microorganism has also been isolated from hundreds of other species (Hopla, 1974). However, the defined role of each one of these species in the ecology of *F. tularensis* is not distinctly defined and might be different and / or restricted to specific geographical areas. Particularly, some confusion about incidental and reservoir hosts should be clarified. Indeed, all incidental hosts are not required for the perpetuation (long term maintenance) of an infectious agent and some incidental animal hosts might only represent a “bridge” for *F. tularensis* between wildlife and humans (Telford and Goethert, 2010).

## Tularemia in animals

Tularemia in animals is extremely complex because of the numerous species described as being susceptible to this disease. Additionally, there might be differences between observations made in natural versus experimental infections, and importantly regarding the epidemiologic relevance of the experimental results in the environment. Despite the countless experiments performed and published describing the development of tularemia in animals, the route of transmission, the organs affected, the progress of lesions and inflammatory host responses as well as the range of animal species acting as reservoir between epizootics need to be clarified with regard to the various *F. tularensis* lineages.

Historically, animal species affected by *F. tularensis* were classified in three groups according to their susceptibility (to infection) and sensitivity (severity of clinical manifestation) (Hopla, 1974; WHO., 2007; Mörner and Addison, 2008): class 1 «acute disease after inoculation of 1–10 bacteria with rapid multiplication in blood and tissues»; class 2 «death after inoculation of 10^8^-10^9^ bacteria; survival may occur at lower doses and then provide immunity» and class 3 «genera resistant to *F. tularensis*» (Hopla, 1974; WHO., 2007). Since this classification is primarily based on the bacterial dose necessary to cause death and the ability to spread *via* the blood and lymphatic streams, it mainly results from observations made after experimental infection. Nevertheless, the natural occurrence, progress of infection, and development of efficient immunity in specific species might also be parameters to consider to enable animal species to be grouped to understand their role in the maintenance and perpetuation of *F. tularensis* in the environment. In future investigations, species highly susceptible and sensitive to *F. tularensis* infection might be considered as incidental hosts, while animal species moderately susceptible or resistant may be considered in terms of their role in the long term maintenance and spread of this bacterium.

Countless rodent species belonging to the Families of *Cricetidae* and *Muridae* are highly susceptible and sensitive to *F. tularensis*. They develop acute tularemia and succumb quickly after infection (Larson, 1945; Ditchfield et al., 1960; Mörner, 1992; Origgi et al., 2015). Several lagomorphs are similarly affected by this disease (Burroughs et al., 1945; Morner et al., 1988b; Park et al., 2009). It is worth mentioning that variabilities in sensitivity to *F. tularensis* among susceptible species are known. For example, the common rabbit (*Oryctolagus cuniculus*) was used to differentiate *F. tularensis* subsp*. tularensis* and *F. tularensis* subsp. *holarctica* and it is generally considered as susceptible but less sensitive to *F. tularensis* subsp. *holarctica* (Sjöstedt, 2015). Although *O. cuniculus* was found to be naturally infected with *F. tularensis* subsp. *holarctica*, more details about the health status of the rabbits and pathological lesions are required (Runge et al., 2011; Lopes de Carvalho et al., 2016). Moreover, the sensitivity of animal species was suggested to be influenced by rodenticides or pesticides released in the environment but this aspect needs confirmation (Vidal et al., 2009; Bandouchova et al., 2011). Sporadic cases or outbreaks in rodents and lagomorphs are frequently reported and these species are without doubt involved in a part of the life cycle of *F. tularensis* in the environment. However, not all species may be incidental hosts and the susceptibility and sensitivity of the different species should be carefully described. The situation in beavers is noteworthy and deserves more attention in the future. Beavers in North America (*Castor canadensis*) are known to be part of the enzootic cycle of *F. tularensis* (Scott, 1940; Jellison et al., 1942) and strains isolated from this animal species belong to *F. tularensis* subsp. *holarctica* (Mörner, 1992; Kugeler et al., 2009). However, only a few strains of *F. tularensis* subsp. *holarctica* were isolated from beavers in Europe (Sissonen et al., 2015; Schulze et al., 2016). *C. fiber* is the native species of beaver in Eurasia and this finding presents questions on the susceptibility of *C. fiber* to *F. tularensis*. *C. canadensis* was intentionally introduced in Eurasia in 1937, more specifically in Finland and populations still exist in an area spanning Finland and Russia (Parker et al., 2012). Interestingly, the two beaver isolates characterized by Sissonen and colleagues are from Finland and were isolated from *Castor* sp. meaning the exact species was not identified. However, the authors stated that those beavers were found in areas where only *C. canadensis* is known to live (Sissonen et al., 2015). Recently, *F. tularensis* subsp. *holarctica* was isolated from the carcass of an Eurasian beaver, *C. fiber*, found in the Berlin/Brandenburg region in Germany (Schulze et al., 2016). *C. fiber* was previously shown to raise antibodies against *F. tularensis* but isolation of the microorganism was not reported (Morner et al., 1988a). Investigations aiming to assess the susceptibility and sensitivity of *C. fiber* to *F. tularensis* should be supported and more precisely, studies evaluating their cause of death, in association with serological studies.

Some other wildlife species of the Order *Soricomorpha* (previously named *Insectivora*) like *Talpa* sp. and the Order *Eulipotyphla* like *Sorex* sp. were also described as being naturally infected with *F. tularensis* (Kohls and Steinhau, 1943; Elashvili et al., 2015). Moreover, some non-human primate species are also considered as very susceptible species and reports of cases due to *F. tularensis* subsp. *tularensis* and *F. tularensis* subsp. *holarctica* in zoos were published (Posthaus et al., 1998; Hoelzle et al., 2004; Abril et al., 2007; Gyuranecz et al., 2009; Ketz-Riley et al., 2009). Occasional infections in birds were reported, mainly from North America but also from Sweden. Birds are generally considered as resistant to *F. tularensis* infection and as having no relevant role as hosts in the epidemiology of this bacterium (reviewed in Mörner, 2008).

Over the last decade, the quantity of studies detecting *F. tularensis* in wildlife animals and in the environment has risen extraordinarily. The increased awareness and number of animals tested, led to isolation of *F. tularensis* from species previously described as not being particularly susceptible to this bacterium e.g., species belonging to the Order Carnivora, like the red fox, *Vulpes vulpes*, or the stone marten, *Martes foina* (Origgi et al., 2013; Schulze et al., 2016). The relevance of these species in the cycle of *F. tularensis* and their epidemiological significance are still to be investigated but might represent single occurrences.

Production and companion animals, other than rodents and lagomorphs, are a particular concern because of their proximity to human beings. Tularemia in the domestic cat (*Felis catus*) is reported in North America. It seems to be mainly associated with *F. tularensis* subsp. *tularensis* but *F. tularensis* subsp. *holarctica* was also isolated from a few feline cases (Baldwin et al., 1991; Woods et al., 1998; Farlow et al., 2001; DeBey et al., 2002; Staples et al., 2006). Feline tularemia has not been reported outside North America. It is important to investigate if cases have been missed in other countries or if feline tularemia is principally due to *F. tularensis* subsp. *tularensis*, which is only circulating in North America, or is a consequence of other still unknown parameters. Dogs (*Canis familiaris*) appear to be more resistant to *F. tularensis* and until recently, most of the sporadic cases of tularemia were reported from North America (Meinkoth et al., 2004). However, a report of tularemia in a dog in Norway was published in 2014. The dog developed clinical manifestations after hunting a mountain hare (*L. timidus*). The case was investigated by serology and a 32-fold increase in titer in 2 weeks was noticed by the authors. However, the case could not be bacteriologically confirmed but *F. tularensis* subsp. *holarctica* was isolated from the bone marrow of the captured mountain hare (Nordstoga et al., 2014). Tularemia outbreaks in sheep (*Ovis aries*) were reported in North America and were associated with *F. tularensis* subsp. *tularensis* (O'Toole et al., 2008). More studies are needed regarding animals used for production as well as companion animals, with particularly emphasis on host specificity of the different subspecies of *F. tularensis*. This is necessary to confirm or refute which animal species can be naturally infected and can act as incidental or reservoir hosts. This information is crucial to understand the maintenance and perpetuation of *F. tularensis* but also to evaluate the real risk for transmission to humans.

## Lineages of *F. tularensis* and animal species

The recent advent of new technologies and improvements in typing systems for *F. tularensis*, led to new perspectives, resulting in better understanding of the epidemiological situation of tularemia in animals. As mentioned above, many studies were performed in the last decade to characterize the population genetics of *F. tularensis* strain collections and some of these included animal strains. Moreover, few case reports comprise the typing of the isolated *F. tularensis*.

*F. tularensis* subsp. *tularensis* is divided into two clades A.I and A.II (Figure 1) (Farlow et al., 2005; Staples et al., 2006; Kugeler et al., 2009). Clade A.I predominates in Eastern North America, but occurs thorough North America, while A.II prevails in Western North America. In the study published by Kugeler et al. 184 animal strains were characterized and the authors observed a non-random distribution of the subspecies of *F. tularensis* in animals. In the case of lagomorph species there was an association with specific lineages (the classification used by Kugeler et al. is based on PFGE and not on canSNPs). Briefly, the strains from lagomorphs (cottontail rabbits and jackrabbits) were principally type A (90%, *n* = 52; total *N* = 58) and 40% of the 52 strains were A.I. Unfortunately, the lagomorph species was only described in 31 cases but still revealed remarkable information. All eight A.I strains were isolated from the eastern cottontail *(S. floridanus)* and the 23 strains isolated from the desert cottontail *(S. audubonii)* were A.II (Kugeler et al., 2009). Strains isolated from hares in Quebec, Canada, were all A.I but the hare species was not defined (Antonation et al., 2015). Six strains isolated from hares in Alaska were also A.I (Hansen et al., 2011). Kugeler and colleagues analyzed 44 feline strains and 41 belonged to the subspecies *tularensis*, with 80% percent of these strains being A.I (Kugeler et al., 2009). Interestingly, another study found that all but one feline isolate tested (total *N* = 27) were A.I (Larson et al., 2014).

Regarding *F. tularensis* subsp. *holarctica*, four clades are described: B.16 (*F. tularensis* subsp. *holarctica* biotype japonica), B.4, B.6, and B.12 (Figures 1, 2) (Svensson et al., 2009b; Karlsson et al., 2013). B.16 is found in Japan and Australia, B.4 is present in Eurasia and North America, B.6 is circulating in North America and Europe and B.12 in Eurasia and North America. Recently, an additional clade of *F. tularensis* subsp. *holarctica* from Tibet, China, was described by Lu et al. (2016). Although strains of *F. tularensis* subsp. *holarctica* are circulating in North America, Eurasia and Australia, a more in depth focus on the lineages of this subspecies was recently described in Eurasia. Unlike *F. tularensis* subsp. *tularensis*, differences in virulence among the lineages of *F. tularensis* subsp. *holarctica* are less clear. So far, a well-defined predilection of specific lineages of *F. tularensis* subsp. *holarctica* for particular host species was not identified (Figure 2). However, some recent observations deserve to be investigated in more detail. *F. tularensis* subsp. *holarctica* was isolated from *L. europaeus* in many countries (Morner et al., 1988b; Gyuranecz et al., 2010; Decors et al., 2011; Müller et al., 2013; Rijks et al., 2013; Nordstoga et al., 2014; Origgi and Pilo, 2016; Hestvik et al., 2017). In Sweden, the first animal tularemia case was diagnosed in 1931 in the mountain hare (Hestvik et al., 2017). Mörner and colleagues also investigated the presence of *F. tularensis* in *L. europaeus* and *L. timidus* from Sweden but they could not detect it in *L. europaeus* (Morner et al., 1988b). The first case in the European brown hare in Sweden was only identified in 2002 (Hestvik et al., 2017). The reason for this late discovery is still unknown and merits further studies. In 2010, Gyuranecz and colleagues published a study describing the pathological lesions due to *F. tularensis* subsp. *holarctica* lineage B.13 in *L. europaeus*. The common finding of this study is a polyserotitis with the pericardium, the lung and the kidney as the main affected organs (Gyuranecz et al., 2010). However, Origgi and Pilo found that the most affected organs in *L. europaeus* infected with *F. tularensis* subsp. *holarctica* lineage B.FTNF002-00 [B.6, subgroup B18, this subgroup is specific to Western Europe (Dwibedi et al., 2016)] were the spleen and the liver (Origgi and Pilo, 2016). It has to be mentioned that the hares investigated by Gyuranecz and colleagues were hunted, while the hares investigated by Origgi and Pilo were terminally-ill. The group of Gyuranecz subsequently performed an experimental infection in Fischer 344 rats with strains belonging to both lineages (Kreizinger et al., 2017). They did not observe variance in pathological lesions due to the strains but found differences in weight loss values, recovery time, and mortality. In the study published by Hestvik and colleagues, the pathology resulting from natural infection with *F. tularensis* subsp. *holarctica* in the mountain hare and in the European brown hare was characterized. They found similar pathological lesions in both hare species. The lineages of the 23 strains of *F. tularensis* subsp. *holarctica* were characterized and seven found to belong to B.6 (subgroup B.7), while 16 were B.12 (basal sublineage B.26, subgroups B.23, B.39, and B.20) and originated from 21 *L. europaeus* and two *L. timidus*. No differences were observed in the pathological lesions due to both lineages (Hestvik et al., 2017). Sissonen and colleagues reported that among the strains analyzed in their study, one strain isolated from *L. timidus* was B.4 and one strain isolated from *L. europaeus* was B.6 sublineage B.7. Additionally, 50 strains were B.12 (24 from *L. timidus* and 26 from *L. europaeus*) (Sissonen et al., 2015).

In Japan, the lineage B.16 (*F. tularensis* subsp. *holarctica* biotype japonica) is often isolated from the japanese hare, *L. brachyurus*. However, a recent report from Turkey identified a strain of *F. tularensis* subsp. *holarctica* B.16 based solely on phenotypic characteristics (Kilic et al., 2013). Moreover, the distribution of this lineage might be more extended than previously thought since *F. tularensis* subsp. *holarctica* biotype japonica was recently isolated in Australia from ringtail possums (*Pseudocheirus peregrinus*) (Eden et al., 2017). Finally, Wang and colleagues published a study including isolates of *F. tularensis* from the Tibetan region identified by canSNP analysis as belonging to the lineage B.16 (Wang et al., 2014). Lu and colleagues subsequently investigated isolates from the same region and some were also identified as B.16. However, these isolates do not ferment glycerol, which is a distinguishing phenotype of *F. tularensis* subsp. *holarctica* biotype japonica. Analysis of region of differences (RD) and MLVA further showed an intermediate position of the Tibetan isolates between those belonging to lineage B.16 and those of the other lineages B.4, B.6, and B.12. The authors therefore proposed to add a major lineage to the subspecies *holarctica* (Lu et al., 2016).

A recent article published by Timofeev and colleagues analyzed 25 strains of *F. tularensis* subsp. *mediasiatica* and compared them to four strains previously analyzed by MLVA. Among those strains, seven were isolated from animals other than arthropods. One strain of M.II was isolated from a Siberian red vole and the six other strains belonged to M.I and were recovered from hares and gerbils (Timofeev et al., 2017).

These findings highlight the necessity to expand and intensify sampling, particularly in Asia and Oceania, to better understand the geographical and animal distribution of the lineages *F. tularensis* subsp. *holarctica* and *F. tularensis* subsp. *mediasiatica*.

## Conclusion

Tularemia in animals is a vast topic and despite the large amount of research performed on *F. tularensis* in animals, many questions are still open. It is clear that rodent and lagomorph species are affected by this microorganism in all countries where *F. tularensis* is present. However, it is still unclear whether these animal species are important for long term maintenance of *F. tularensis* in the environment. In fact, details on which species are incidental or reservoir hosts are still to be defined with more precision. In this respect, serological methods are important and give useful information but for a full interpretation of the results, complementary investigations should be performed. Positive serological results inform about the infection of an animal with *F. tularensis* but does not necessarily imply the development of clinical manifestations or determine the potential shedding of *F. tularensis*. It would therefore be important when possible to couple serological studies with field observations and / or extra sampling to clarify these aspects in the future. Furthermore, the route of infection, the progress of the infection within different hosts, the host immune response of specific animal species and the role of ecological factors also merits more attention. Another noteworthy parameter to consider is that animal species belonging to other Orders than Rodentia and Lagomorpha are most of the time ignored in studies and during routine diagnostics. Additionally, the complexity of the ecology of *F. tularensis* means short studies may only reflect a partial picture of the situation and sustained and coordinated efforts will be required to unravel particularly striking observations. For example, epizootics or sporadic cases “appear” and “disappear,” sometimes without implementation of specific measures to control the disease (Dobay et al., 2015). This simple observation can raise a multitude of questions: Does the source of infection exist for a limited time? Do animal populations develop immunity? Are there major individual variations in terms of susceptibility and sensitivity? Are biological and / or physical parameters involved? If yes, how and to which degree? This shows how little information is available and gives a modest insight into the questions that need to be answered.

The recently developed rapid sequencing technologies open novel perspectives for rational design of studies and are a great opportunity to better understand the ecology and epidemiology of *F. tularensis* in distinct geographical areas. Phylogenetic data of *F. tularensis* in animals is becoming available but is still fragmented and needs to be confirmed. The molecular tools available today are rapid and safe. Moreover, they allow a low or high resolution of typing and even enable the possibility to mix both depending on the questions to answer. For routine diagnostic laboratories, the most important analysis should lead to the confirmation of presence or absence of *F. tularensis*. These laboratories usually do not attempt to cultivate *F. tularensis* because of safety concerns and a very high resolution of typing is not always necessary. However, if a case is interesting enough for publication, authors might consider including both the animal species and the lineage of *F. tularensis*. A technical aspect deserving more development and extensive validation is the application of PCR for typing directly from clinical specimens. The establishment of protocols for typing without the requirement of previous cultivation of *F. tularensis* would be a useful development for diagnostic laboratories.

In summary, a large body of information is available about tularemia in animals, in particular concerning the animal species that are mostly affected. However, the numerous animal host species and number of subspecies of *F. tularensis* complicates the understanding of the biology in specific environments. More precision is needed in the definitions used to describe studies and the integration of several disciplines is crucial to overcome the complexity of *F. tularensis*. The development of methods to discriminate strains can add data to better understand variations in the ecology of this bacterium and research is currently being performed in this field. Additionally, extensive sampling including full documentation would be very informative about the evolution and genetic diversity of *F. tularensis* in animals. Hence, detailed field, clinical, pathological and laboratory information is crucial to subsequently test experimentally functional hypotheses on the spread and life style of *F. tularensis*.

## Author contributions

The author confirms being the sole contributor of this work and approved it for publication.

### Conflict of interest statement

The author declares that the research was conducted in the absence of any commercial or financial relationships that could be construed as a potential conflict of interest.
